# Visuospatial attention in the lateralised brain of pigeons – a matter of ontogenetic light experiences

**DOI:** 10.1038/s41598-017-15796-6

**Published:** 2017-11-14

**Authors:** Sara Letzner, Onur Güntürkün, Stephanie Lor, Robert Jan Pawlik, Martina Manns

**Affiliations:** 1 Biopsychology, Institute of Cognitive Neuroscience, Ruhr-University Bochum, 44780 Bochum, Germany; 2 Cognitive Neurophysiology, Department of Child and Adolescent Psychiatry, Faculty of Medicine, TU Dresden, 01307 Dresden, Germany; 3 Institute of Medical Psychology and Behavioral Immunobiology, University Hospital Essen, University of Duisburg-Essen, 45122 Essen, Germany

## Abstract

The ontogenetic mechanisms leading to complementary hemispheric specialisations of the two brain halves are poorly understood. In pigeons, asymmetrical light stimulation during development triggers the left-hemispheric dominance for visuomotor control but light effects on right-hemispheric specialisations are largely unknown. We therefore tested adult pigeons with and without embryonic light experience in a visual search task in which the birds pecked peas regularly scattered on an area in front of them. Comparing the pecking pattern of both groups indicates that the embryonic light conditions differentially influence biased visuospatial attention under mono- and binocular seeing conditions. When one eye was occluded, dark-incubated pigeons peck only within the limits of the visual hemifield of the seeing eye. Light-exposed pigeons also peck into the contralateral field indicating enlarged monocular visual fields of both hemispheres. While dark-incubated birds evinced an attentional bias to the right halfspace when seeing with both eyes, embryonic light exposure shifted this to the left. Thus, embryonic light experience modifies processes regulating biased visuospatial attention of the adult birds depending on the seeing conditions during testing. These data support the impact of light onto the emergence of functional dominances in both hemispheres and point to the critical role of interhemispheric processes.

## Introduction

Examples from various vertebrate and invertebrate species indicate a profound fitness advantage when brains display a division of labour between the two brain halves^1–4^. Lateralised individuals of a species show enhanced sensorimotor and cognitive performances^5–9^, are better in conducting two tasks in parallel^10–14^, and display efficient interhemispheric cooperation^15,16^. Thereby, the hemispheres differ in their modes to analyse and/or to evaluate information that in turn may lead to hemispheric dominances for specific functions. In vertebrates, the left hemisphere typically controls routine behaviour, fine-tuned discrimination and categorisation, or recognition of conspecific vocalisation while the right hemisphere dominates the early detection of unexpected stimuli, behaviour in emergency situations, or spatial cognition^17,18^.

The development of hemispheric asymmetries is controlled both by genes and environment^19–24^ but we only marginally understand in which way the emergence of left- and right-hemispheric specialisations depend on genetic, environmental and epigenetic interactions. The visual system of birds is a well-established model to unravel the neuronal processes leading to a lateralised brain. As the optic nerves cross virtually completely, hemispheric specialisations can be easily tested just by temporarily occluding one eye with an eye cap. Behavioural asymmetries are related to structural and neurophysiological left-right differences in the visual pathways. In chicks and pigeons, the development of visual asymmetries depends on asymmetrical light stimulation during ontogeny^15,20,21,25,26^. Owing to the asymmetrical position of the embryo within the egg, the right eye is close to the translucent eggshell and the left eye is occluded by the body. Consequently, light shining through the eggshell stimulates the right eye more intensely than the left one, which leads to asymmetrical activity-dependent differentiation processes culminating in a left-hemispheric dominance for visuomotor control^20,25^. Dark-incubation of avian embryos prevents the development of visual asymmetries^26,27^ and monocular deprivation before^28^ or after hatching^29^ reverses the normal pattern. In pigeons, comparison of left- and right-hemispheric performances suggests that left-hemispheric dominances result from a light-dependent modulation of neuronal circuits onto both hemispheres^14,27,30,31^. This indicates a crucial impact of interhemispheric interactions^20,32^ and raises the possibility that right-hemispheric specialisations are determined in parallel.

A typical right-hemispheric specialisation is spatial attention. In humans, lateralised attention control is shown in a visuospatial bias to the left hemispace in line-bisection^33^, or cancellation tasks^34^ and enhanced right-hemispheric activation is supported by neuro-imaging and neurophysiological studies^35^.

Birds display a similar leftward bias in their pecking activity in a food detection task in which they are required to explore an area uniformly spread with grains^36,37^. This pattern suggests a right-hemispheric dominance for controlling visuospatial attention^37^. Experiments with chicks provide evidence for a light-dependent development^36,38^ and therefore support a causal relation between the emergence of left- and right-hemispheric dominances. A similar pattern could be expected in pigeons but must be verified since both species display profound differences in the ontogeny of visual asymmetries^25,39^. Additionally, it might be intriguing to analyse visual attention under monocular seeing conditions. Pigeons are birds with laterally placed eyes so that the right hemisphere processes primarily information from the left, and the left hemisphere processes information from the right visual field with only a limited binocular overlap^40^. Dominance and hence, enhanced activity of the right hemisphere in a task requiring visuospatial attention might also become obvious as differentially biased search strategies when seeing with the left or right eye. Moreover, it is conceivable that attentional processes differ between mono- and binocular seeing conditions^41^ and hence, might be differentially modified by the embryonic light conditions^16^.

We therefore compared the visuospatial attention pattern in pigeons with and without embryonic light experience under different seeing conditions to explore the light-dependent development of right hemispheric dominances.

## Results

### Pecking Pattern under Monocular Seeing Conditions

**Figure 1:**
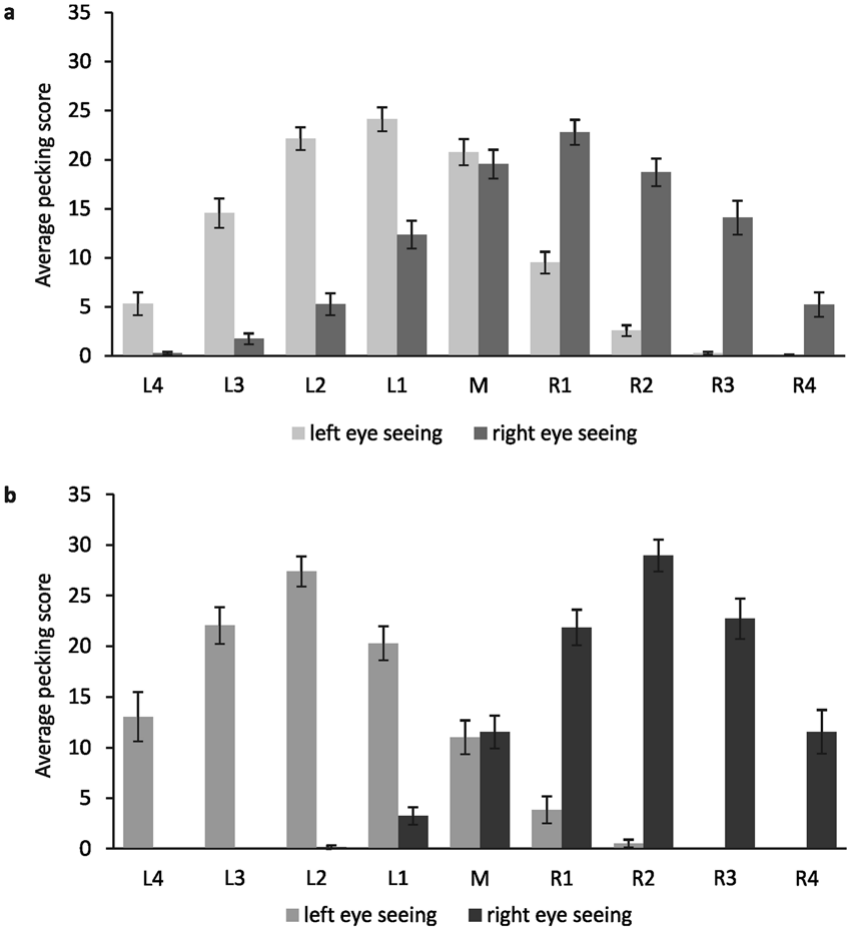
Mean pecking scores under left- and right eye seeing conditions in pigeons with (**a**) and without (**b**) embryonic light experience. Scores indicate the order in which pigeons pecked in every column on the left (L1–L4), middle (M), and right (R1-R4). Bars represent standard errors.

**Figure 2:**
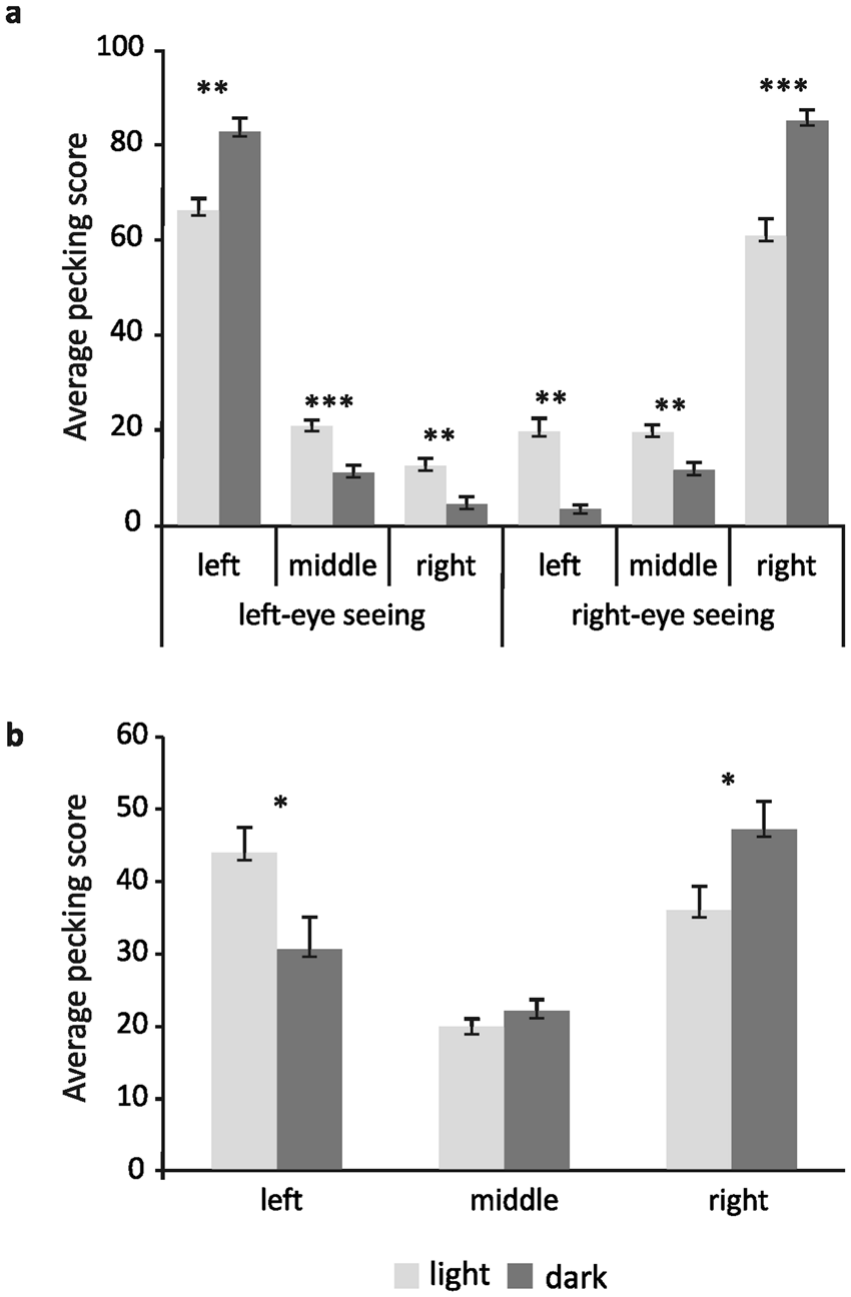
Summed up pecking scores into the left, middle or right halfspace of pigeons with and without embryonic light experience under mono- (**a**) and binocular (**b**) seeing conditions. Bars represent standard errors (*P < 0.05, **P < 0.01, ***P < 0.001 according to t-tests for independent samples).

When one eye was occluded, pigeons centred their pecks into the hemifield of the seeing eye (Fig. 1). In contrast to light-exposed individuals (Fig. 1a), dark-incubated pigeons barely pecked across the midline and completely neglected the lateral columns of the contralateral field (Fig. 1b). For comparison of the differential pecking pattern, we combined pecking scores of the left and right columns (Fig. 2a) and analysed them using a 2 × 2 × 3 mixed analysis of variance (MANOVA) with the between-subjects factor “*Group*” (dark-incubated animals, light-exposed animals) and the two within-subject factors “*Field*” (left, middle, right) and “*Seeing Condition*” (left eye, right eye).

**Figure 3:**
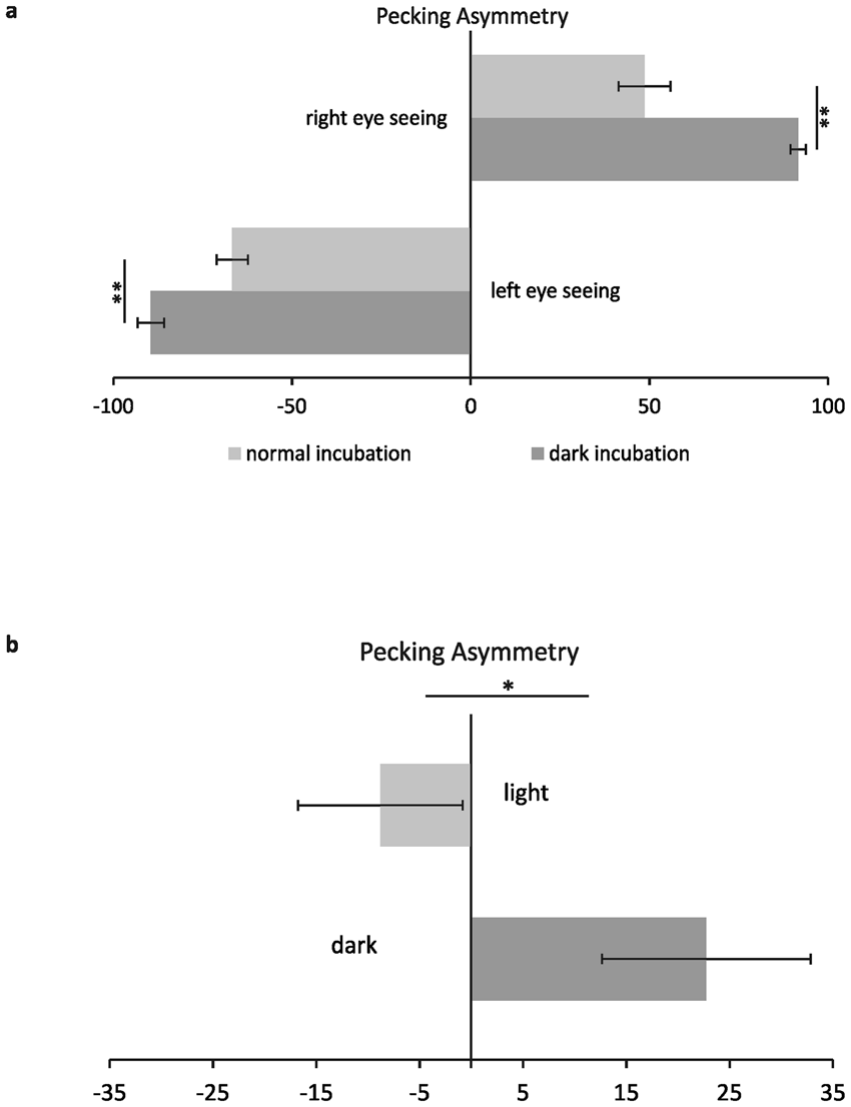
Mean pecking asymmetry of pigeons with and without embryonic light experience under mono- (**a**) and binocular (**b**) seeing conditions. Bars represent standard errors (*P < 0.05, **P < 0.01, according to t-tests for dependent or independent samples).

Statistical analysis confirmed that the preferential pecking field depended on the seeing condition (“*Seeing condition* × *Field*” interaction: F_(2,118)_ = 873.85; p < 0.001; partial η^2^ = 0.94) but was also influenced by the factor “*Group”* as indicated by significant interaction “*Seeing Condition* × *Field* × *Group”* (F_(2,118)_ = 57.19; p < 0.001; partial η^2^ = 0.49). Posthoc comparisons verified that dark-incubated pigeons displayed highest pecking scores into the field ipsilateral to the seeing eye while light-exposed pigeons pecked more often onto the midline and into the contralateral hemifield (Fig. 2a). There was however, no difference in the pecking scores between the seeing conditions irrespective of the group (“*Seeing Condition* × *Group*” interaction: F_(1,59)_ = 0.62; p = 0.43; partial η^2^ = 0.01). Accordingly, the degree of pecking asymmetry did not differ between the seeing conditions in neither group (Fig. 3a). In dark-incubated animals however, pecking asymmetry was significantly more pronounced than in the light-exposed group (left-eye asymmetry: t = −3.32, p < 0.01; right-eye asymmetry: t = 4.09, p < 0.001; Fig. 3a).

### Pecking Pattern under Binocular Seeing Conditions

**Figure 4:**
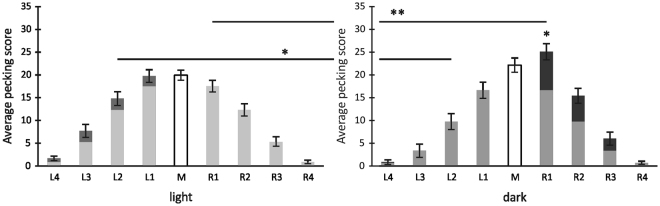
Mean pecking scores for pigeons with and without embryonic light experience under binocular seeing conditions. Scores indicate the order in which pigeons pecked in every column of the left (L1–L4), middle (M), and right (R1–R4) field. Highlighted is the difference between the order of left and right pecks. Bars indicate standard errors (*P < 0.05, **P < 0.01 according to posthoc t-tests for dependent or independent samples).

Both groups centred their pecks around the midline but pecking scores onto left- or right-sided columns differed (Fig. 4). These differences were analysed by a 2 × 2 × 4 MANOVA with the between-subjects factor “*Group*” (dark-incubated animals, light-exposed animals) and the two within-subject factors “*Side*” (left, right) and “*Column*” (1–4).

We observed a significant main effect of “*Column*” (F_(3,174)_ = 215.25; p < 0.001; partial η^2^ = 0.79) indicating that in general, animals showed a tendency to peck closer to the midline (Fig. 4). This effect was modulated by “*Side*” and “*Group*” as indicated by significant interactions between “*Side* × *Group*” (F_(1,58)_ = 4.84; p < 0.05; partial η^2^ = 0.08) and “*Side* × *Column* × *Group*” (F_(3,174)_ = 3.82.; p < 0.05; partial η^2^ = 0.06). Posthoc comparisons verified that dark-incubated pigeons pecked significantly more into the right (t-test for independent samples: t = 2,256, p < 0.05), light-exposed pigeons more into the left hemifield (t-test for independent samples: t = −2,076, p < 0.05, Fig. 2b). Accordingly, pecking asymmetry scores differed significantly between the two groups (t-test for independent samples: t = 2,363, p < 0.05, Fig. 3b).

Both groups included individuals displaying a left- or a right-side bias in their binocular pecking pattern (for individual data see Supplementary Material SI 1). We therefore correlated the binocular pecking asymmetry score with the monocular ones to estimate potential interrelations between mono- and binocular performances. Whereas neither the left- nor the right-eye pecking asymmetry could be correlated with the binocular pecking bias in dark-incubated pigeons, we detected a significant correlation between the binocular and the right-eye pecking asymmetry in light exposed pigeons (R = 0.55, p < 0.05; for all data see Supplementary Material SI 2).

## Discussion

Our results demonstrate that embryonic light experience in pigeons profoundly alters visuospatial attention as revealed in pecking patterns in a food cancellation task. Our data further show that this effect differs for bi- and monocular visual seeing conditions. This overall pattern provides important insights into the processes that generate the lateralised functional organisation of the brain. We will discuss our data, one by one.

In accordance with previous studies^37,42^ but in contrast to other bird species^43^, our light-exposed pigeons displayed a preference to peck into the left hemifield when seeing with both eyes. Dark-incubated pigeons on the other hand, displayed a significant bias to the right hemifield, which indicates that pigeons without embryonic light experience develop a left hemispheric dominance for visuospatial attention. Thus, light incubation does not induce an asymmetry of visuospatial asymmetry: It is already present and induces a right hemifield bias. What light incubation does is to shift visuospatial attentional asymmetry towards the left hemifield. This is in contrast to the described absence of an attentional bias in dark-incubated chicks^36,38^ but adds to reports showing light-independent hemispheric asymmetries in pigeons^32^, and chicks^38,44–47^. Since our light-exposed group however, consisted of animals showing individual left- or right-hemispheric dominances, other genetic, environmental and epigenetic factors^19^ including age- or stress-related influences^33,48–51^ might interact with light.

Pigeons are birds with laterally placed eyes and small overlapping visual fields^52^. Thus, when tested with one eye occluded, visuospatial attention should primarily be centred to the hemifield ipsilateral to the seeing eye. But different from dark-incubated individuals, light-exposed pigeons pecked significantly more into the contralateral hemifield. Thus, both brain hemispheres of light-exposed birds have to have attentional fields that encompass also parts of the visual field of the other hemisphere. In order to understand the neuronal mechanisms that could mediate such a pattern, we have to differentiate between effects onto mono- and binocular performances^20,40^.

Owing to the asymmetrical position in the egg, the right eye is more intensely activated by light shining through the eggshell. Accordingly, embryonic visual stimulation should primarily affect activity-dependent differentiation of the right eye/left hemisphere system^20,40,53,54^. But enhanced right-eye stimulation does not simply increase differentiation processes within the stronger activated left hemisphere. Monocular modulation of retinal activity has profound effects onto both brain sides as indicated by experiments with monocular injections of the sodium channel blocker TTX and the neurotrophic factor BDNF^30,31^. These effects could be mediated by commissural systems at midbrain level, especially since these systems are presumably involved in attentional control, thereby integrating retinal bottom-up and forebrain top-down information^55,56^.

Apart from effects onto visual field size of each hemisphere, enhanced embryonic stimulation of the right eye also affects attentional control under binocular seeing conditions. Accordingly, we observed a correlation between right-eye and binocular attentional asymmetry. Pigeons with a diminished right side bias when seeing with the right eye showed a larger attentional shift to the left hemifield with both eyes. A similar interrelation between the right-eye and binocular performance has been observed in a food discrimination task showing that a stronger right eye/ left-hemispheric dominance for visuomotor control is related to an enhanced binocular performance^7^.

It is conceivable that interhemispheric systems^55,57^ also mediate the underlying bilateral effects. Their crucial role presumably persists into adulthood as flexible systems that regulate interhemispheric cooperation or inhibition depending on the situational context and/or current seeing conditions^20,40,41^. This means that biased visuospatial attention is possibly not hard-wired but depends on the balance of left- and right-hemispheric network activity – an idea that is also suggested in human research. Studies in neglect patients and control subjects indicate a right hemispheric dominance in controlling spatial attention, while brain imaging studies support symmetrically organized dorsal fronto-parietal attention networks^58^. Therefore, only lateralised interactions between relevant brain structures lead to hemispheric asymmetries and not lateralisation of spatial attention *per se* ^58,59^.

Apart from the current seeing conditions, the interplay between focused and global attentional processes might also influence the observed attentional pattern. In vertebrates, the left hemisphere is specialised to focus attention to specific targets or cues for controlling learnt routine behaviour. The right hemisphere on the other hand, controls broad attention to detect unexpected or novel stimuli^60^. Pecking seeds as in the cancellation task is a well-established visuomotor behaviour in pigeons. Accordingly, it is conceivable that the attentional bias to the right hemifield in dark-incubated pigeons reflects the left hemispheric dominance on focused attention during feeding. The observed shift to the left hemifield in light-experienced pigeons might be the consequence of processes that enhance the impact of right-hemispheric processes. This idea might also explain differences in the attentional pattern of dark-incubated chicks and pigeons. Differences might be related to the differential developmental stages of adult pigeons and young chicks (e.g.^25^). The absence of an attentional bias in dark-incubated chicks^36,38^ may reflect their less controlled pecking activity^15,38,45^ that develops after embryonic light stimulation and depends on efficient interhemispheric interactions^15,16^. Consequently, attentional pattern may change during development in chicks, too.

In sum, our data further support that the ontogenetic light conditions change the balance of left- and right-hemispheric dominances in the pigeon brain. Asymmetrical visual experience is critically involved in the generation of a left-hemispheric dominance for visuomotor control^27^, reverses superior access to interhemispheric visual information from the right to the left hemisphere^32^ and shifts dominance for attentional control from the left to the right hemisphere. The opposing allocation of functional dominances during ontogeny presumably requires interhemispheric mechanisms, which also enable flexible shifts in lateralised processing depending on the current environmental conditions in the adult brain.

## Methods

### Subjects

Behavioural testing was performed with two experimental groups of adult pigeons (*Columba livia*) of undetermined sex: 40 light-exposed and 20 dark-incubated pigeons. The light-exposed pigeons were obtained from local breeders. The dark-incubated animals stem from lab-own breeding pairs^32^.

The birds were housed individually and placed on a 12/12 h light/dark cycle. Animals were maintained on 85–90% of their free feeding weight throughout the experiments. Food was provided during the experiment and after experimental sessions.

### Apparatus

**Figure 5:**
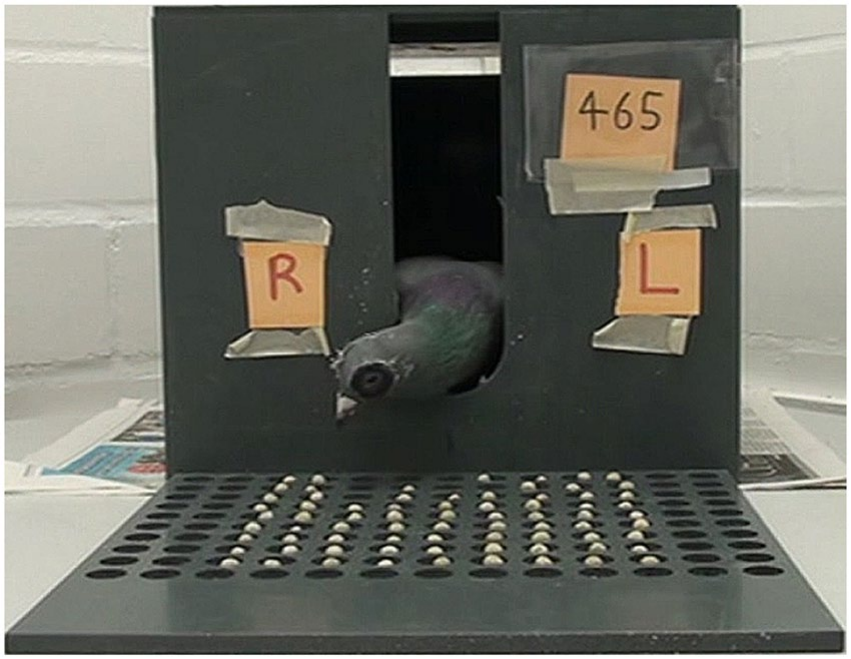
Experimental setup: 81 (9 × 9) peas are homogenously distributed in cavities on a plastic board. The pigeon seeing with both eyes protruded its head through the hole in the box and turned to the right (R) when starting to peck the peas.

The apparatus consisted of a grey plastic box (30 cm wide × 30 cm high × 30 cm deep) with a 7 cm wide hole in the middle of the front panel. The box was located in a chamber with completely white walls. In front of the box a square board was placed (25 cm × 25 cm) with 13 × 10 round cavities. In 9 × 9 of these cavities a single pea was placed (Fig. 5)^37^. The behaviour of the animals was recorded with a video-camera (Sony DCR-SR210) positioned in front of the experimental apparatus.

### Procedure

The animals of the different experimental groups were first positioned inside the box and habituated to the experimental setup to protrude their head through the window in the front panel to directly start pecking grains (Fig. 5). Pigeons did this quiet spontaneously as the box was dark and the experimental room was illuminated. Habituation procedure was repeated three times for 10 min. During testing-phase, the pigeons were placed inside the box and their behaviour was video recorded for a total of 5 min while they were free to peck the peas, in the 9 × 9 array as described above. Each pigeon was tested three times under each seeing condition (binocular, left eye uncovered, right eye uncovered) and mean scores were determined for each condition. Depending on their motivation, the pigeons were tested up to three times per day (each seeing condition once) whereby the order of testing was balanced between birds and days. For monocular testing, one of the pigeon’s eyes was temporally covered by a cardboard patch. To this end, a Velcro-ring was adhered to the feathers around the eye with non-toxic glue. A patch could then be gently velcroed over either eye^41^.

### Analysis

Video recordings were analysed on a computer using the windows media player (Windows 7). For this analysis, the surface of the board was divided into nine vertical columns: the central midline (CTR), four left (L1, L2, L3, L4) and four right (R1, R2, R3, R4) columns and the number of pecks into each column was counted (Diekamp *et al*., 2005). Pigeons made only a few pecks, and after each successful peck, their attention shifted to the areas where grains remained so that the amount and localisation of remaining grains interferes with endogenous attention. To include this factor in our analysis, the spatial position of the first 10 pecks was scored based on the order in which they occurred, with the first peck given the highest score of 10, and normalized to the summation of the weighted pecks. Every peck was counted independently whether it was successful or not, however, repeated pecking in one cavity was counted as only one peck.

Individual pecking asymmetry (PA) was determined by summing up the scores for all four columns for each side using the following formula:

$${\rm{PA}}=[({\rm{R}}-{\rm{L}})/({\rm{R}}-{\rm{L}})]\times 100$$

R = score for the right field, L = score for the leftfield.

The statistical analysis was performed with Statistica 13 (Dell Inc.). Normal distribution was evaluated by Kolmogorov–Smirnov and Shapiro-Wilk tests and homogeneity of variance by Levene as well as Brown-Forsythe-tests. Pecking scores were analysed by running mixed repeated measures analysis of variance. For post-hoc comparisons dependent and independent t-test were conducted. Correlations were estimated by Peason’s *r*. Apart from comparing all animals from both experimental groups, we randomly selected about 20 pigeons of the light-exposed group using the case selection tool of the IBM SPSS 20 Statistic package. We repeated case selection five times and compared the five light-exposed groups with the dark-incubated pigeons repeating all statistical analyses. Statistical data are summarised in Supplementary Materials SI 3 and 4.

### Ethical statement

The study was carried out in compliance with the European Communities Council Directive of November 24, 1986 (86/609/EEC) and the specifications of the German law for the prevention of cruelty to animals, and was approved by the animal ethics committee of the Landesamt für Natur, Umwelt und Verbraucherschutz NRW, Germany. All efforts were made to minimise the number of birds used and to minimize suffering.

### Data Availability

The datasets generated during and/or analysed during the current study are available from the corresponding author on reasonable request.

## Electronic supplementary material

Supplementary Information
