# Supplementary material for: Visuospatial attention in the lateralised brain of pigeons – a matter of ontogenetic light experiences
Source: Sci Rep. 2017 Nov 14;7:15547. doi: 10.1038/s41598-017-15796-6 (PMC5686156; doi:10.1038/s41598-017-15796-6)
Supplement: Supplementary file 1 — Supplementary Information [file 41598_2017_15796_MOESM1_ESM.pdf]

SUPPLEMENTARY INFORMATION

Visuospatial attention in the lateralised brain of pigeons – a matter of ontogenetic light experiences

(Running head: Visuospatial attention in the lateralised brain of pigeons)

Sara Letzner[1,2], Onur Güntürkün[1], Stephanie Lor[1], Robert Jan Pawlik[3], Martina Manns[1*]

[1] Biopsychology, Institute of Cognitive Neuroscience, Ruhr-University Bochum, 44780 Bochum, Germany

[2] Cognitive Neurophysiology, Department of Child and Adolescent Psychiatry, Faculty of Medicine, TU Dresden, Germany

[3] Institute of Medical Psychology and Behavioral Immunobiology, University Hospital Essen, University of Duisburg-Essen, Germany

[*]Corresponding author: Martina Manns, martina.manns@rub.de

Visuospatial attention in the lateralised brain of pigeons

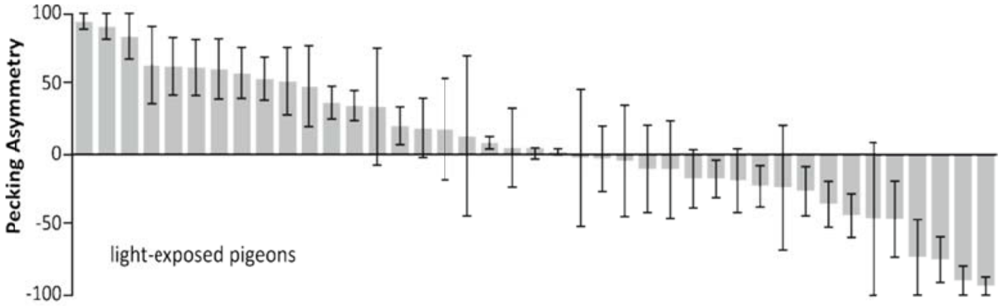

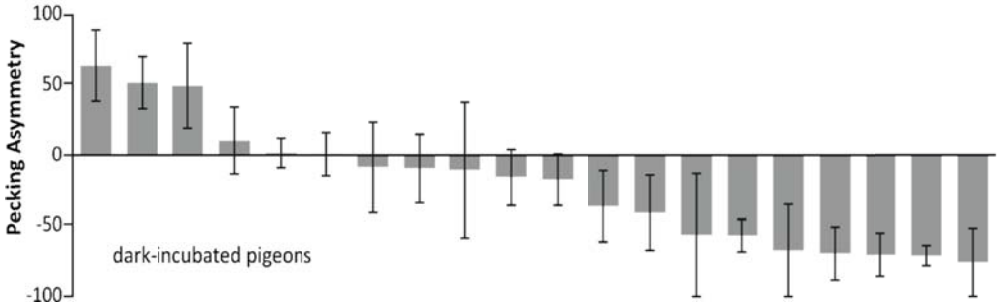

SI 1: Individual pecking asymmetry of animals with and without embryonic light experience under binocular seeing conditions. Positive values indicate a bias to the left, negative values to the right hemispace (bars indicate standard errors).

Visuospatial attention in the lateralised brain of pigeons

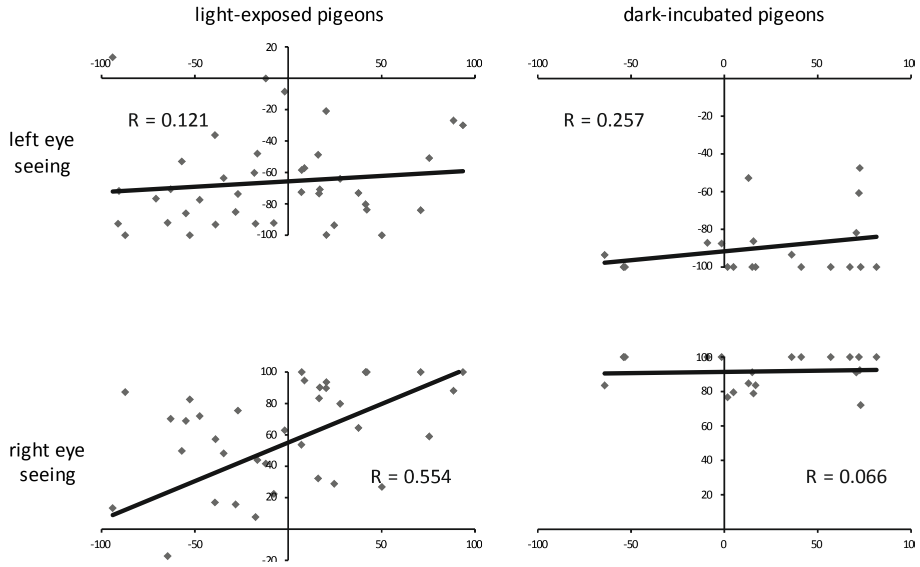

SI 2: Pearson correlation between pecking asymmetry under bi- (x-axis) and monocular (y-axis) seeing conditions. Positive values indicate an attentional bias to the right and negative values to the left hemispace.

Visuospatial attention in the lateralised brain of pigeons

Table 1: Binocular Data: MANOVAs with randomly selected individuals of the light-exposed (LE) and all light-deprived (LD) pigeons

	Original data set<br>LE n= 40<br>LD n=20	Selection 1<br>LE n= 20<br>LD n=20	Selection 2<br>LE n= 20<br>LD n=20	Selection 3<br>LE n= 16<br>LD n=20	Selection 4<br>LE n= 18<br>LD n=20	Selection5<br>LE n= 22<br>LD n=20
2×2×4 MANOVA with the between-subjects factor "*Group*" (light-deprived animals, light-exposed animals) and the two within-subject factors "*Side*" (left, right) and "*Column*" (1-4)						
Group	$F_{(1,58)}=1.314$; p=0.256	$F_{(1,38)}=0.427$; p=0.517	$F_{(1,38)}=1.361$; p=0.251	$F_{(1,34)}=4.848$; p=0.035	$F_{(1,36)}=0.230$; p=0.634	$F_{(1,40)}=1.943$; p=0.171
Side	$F_{(1,58)}=0.616$; p=0.436	$F_{(1,38)}=0.551$; p=0.462	$F_{(1,38)}=0.909$; p=0.347	$F_{(1,34)}=0.156$; p=0.696	$F_{(1,36)}=1.231$; p=0.274	$F_{(1,40)}=0.450$; p=0.506
Side x Group	$F_{(1,58)}=4.842$; p=0.032	$F_{(1,38)}=3.822$; p=0.058	$F_{(1,38)}=15.017$; p=0.0004	$F_{(1,34)}=3.964$; p=0.055	$F_{(1,36)}=2.413$; p=0.063	$F_{(1,40)}=3.633$; p=0.064
Column	$F_{(3,174)}=215.25$; p<0.0000	$F_{(3,114)}=184.664$; p<0.0000	$F_{(3,114)}=184.664$; p<0.0000	$F_{(3,102)}=114.669$; p<0.0000	$F_{(3,108)}=193.116$; p<0.0000	$F_{(3,120)}=178.466$; p<0.0000
Column x Group	$F_{(3,174)}=2,48$; p=0.063	$F_{(3,114)}=1.099$; p=0.353	$F_{(3,114)}=154.029$; p=0.09	$F_{(3,102)}=5.615$; p=0.001	$F_{(3,108)}=0.390$; p=0.760	$F_{(3,120)}=2.812$; p=0.042
Side x Column	$F_{(3,174)}=2.055$; p=0.108	$F_{(3,114)}=2.516$; p=0.061	$F_{(3,114)}=1.009$; p=0.392	$F_{(3,102)}=2.483$; p=0.065	$F_{(3,108)}=1.588$; p=0.197	$F_{(3,120)}=1.503$; p=0.217
Side x Column x Group	$F_{(3,174)}=3.82.$; p=0.011	$F_{(3,114)}=1.963$; p=0.124	$F_{(3,114)}=0.392$; p=0.0004	$F_{(3,102)}=2.321$; p=0.08	$F_{(3,108)}=2.492$; p=0.064	$F_{(3,120)}=3.863$; p=0.011
2×3 MANOVA with the between-subjects factor "*Group*" (light-deprived animals, light-exposed animals) and the within-subject factors "*field*" (left, middle, right)						
Group	$F_{(1,58)}=0.625$; p=0.432	p=1	p=1	$F_{(1,34)}=1.405$; p=0.244	p=1	p=1
Field	$F_{(2,116)}=32.232$; p=0.0000	$F_{(2,76)}=10.08$; p=0.0000	$F_{(2,76)}=13.817$; p=0.0000	$F_{(2,68)}=10.107$; p<0.0000	$F_{(2,72)}=10.839$; p<0.0000	$F_{(2,80)}=11.199$; p<0.0000
Group x Field	$F_{(2,116)}=3.409$; p=0.036	$F_{(2,76)}=3.542$; p=0.034	$F_{(2,76)}=13.441$; p<0.0000	$F_{(2,68)}=4.037$; p=0.022	$F_{(2,72)}=2.228$; p=0.115	$F_{(2,80)}=3.494$; p=0.035
Asymmetry of attentional bias (t-test)	LE: 8.805<br>LD: -22.747<br>t= 2.363 p = 0.022	LE: 8.753<br>LD: -22.747<br>t= 2.096 p = 0.043	LE: 31.351<br>LD: -22.747<br>t= 3.999 p = 0.0003	LE: 12.043<br>LD: -22.747<br>t= 2.122 p = 0.041	LE: 3.501<br>LD: -22.747<br>t= 1.710 p = 0.096	LE: 8.207<br>LD: -22.747<br>t= 2.009 p = 0.052

SI 3: Random selection was done by using the case selection tool of the IBM SPSS Statistics 20 package. Factors indicating potentially group differences in attentional bias are highlighted in grey (significant factors are highlighted in red, trends in orange).

Visuospatial attention in the lateralised brain of pigeons

Table 2: Monocular Data: MANOVAs with randomly selected individuals of the light-exposed (LE) and all light-deprived (LD) pigeons

	Original data set<br>LE n= 40<br>LD n=20	Selection 1<br>LE n= 20<br>LD n=20	Selection 2<br>LE n= 20<br>LD n=20	Selection 3<br>LE n= 16<br>LD n=20	Selection 4<br>LE n= 18<br>LD n=20	Selection5<br>LE n= 22<br>LD n=20
2×2×3 MANOVA with the between-subjects factor "*Group*" (light-deprived animals, light-exposed animals) and the two within-subject factors "*Field*" (left, middle, right) and "*Seeing Condition*" (left eye, right eye)						
Group	$F_{(1,59)}$=0.62; p=0.433	$F_{(1,38)}$=1; p=0.324	$F_{(1,38)}$=0.07; p=0.797	$F_{(1,34)}$=0.01; p=0.916	$F_{(1,36)}$=0.03; p=0.853	$F_{(1,40)}$=1.10; p=0.3
Seeing Condition	$F_{(1,59)}$=2.51; p=0.119	$F_{(1,38)}$=1; p=0.324	$F_{(1,38)}$=1.93; p=0.173	$F_{(1,34)}$=1.87; p=0.180	$F_{(1,36)}$=1.90; p=0.177	$F_{(1,40)}$=1.10; p=0.3
Seeing Condition x Group	$F_{(1,59)}$=40.62; p=0.433	$F_{(1,38)}$=1; p=0.324	$F_{(1,38)}$=0.07; p=0.797	$F_{(1,34)}$=0.01; p=0.916	$F_{(1,34)}$=0.03; p=0.853	$F_{(1,40)}$= 1.10; p=0.3
Field	$F_{(2,118)}$=59.33; p<0.0000	$F_{(2,76)}$=61.34; p<0.0000	$F_{(2,76)}$=70.47; p<0.0000	$F_{(2,68)}$=41.89; p<0.0000	$F_{(2,72)}$=61.12; p<0.0000	$F_{(2,80)}$=50.38; p<0.0000
Field x Group	$F_{(2,118)}$=4.68; p=0.011	$F_{(2,76)}$=3.91; p=0.0241	$F_{(2,76)}$=15.34; p<0.0000	$F_{(2,68)}$=2.91; p=0.061	$F_{(2,72)}$=4.54; p=0.014	$F_{(2,80)}$=4.51; p=0.014
Seeing Condition x Field	$F_{(2,118)}$=873.85; p<0.0000	$F_{(2,76)}$=786.20; p<0.0000	$F_{(2,76)}$=651.54; p<0.0000	$F_{(2,68)}$=476.80; p<0.0000	$F_{(2,72)}$=723.81; p<0.0000	$F_{(2,80)}$=681.78; p<0.0000
Seeing Condition x Field x group	$F_{(2,118)}$=57.19; p<0.0000	$F_{(2,76)}$=48.59; p<0.0000	$F_{(2,76)}$=55.07; p<0.0000	$F_{(2,68)}$=38.73; p<0.0000	$F_{(2,72)}$=44.36; p<0.0000	$F_{(2,80)}$=48.15; p<0.0000
Asymmetry of attentional bias left - eye seeing (t-test)	LE: 55.712<br>LD: 89.552<br>t= -3.318 p = 0.002	LE: 68.819<br>LD: 89.552<br>t= -2.962 p = 0.005	LE: 80.545<br>LD: 89.552<br>t= -1.765 p = 0.085	LE: 64.719<br>LD: 89.552<br>t= -2.881 p = 0.007	LE: 65.917<br>LD: 89.552<br>t= -3.442 p = 0.0015	LE: 67.506<br>LD: 89.552<br>t= -2.986 p = 0.005
Asymmetry of attentional bias right - eye seeing (t-test)	LE: -48.574<br>LD: -91.661<br>t= 4.087 p = 0.0001	LE: -47.419<br>LD: -91.661<br>t= 4.117 p = 0.0002	LE: -29.202<br>LD: -91.661<br>t= 5.691 p < 0.0000	LE: -43.030<br>LD: -91.661<br>t= 3.862 p = 0.0005	LE: -53.730<br>LD: -91.661<br>t= 3.887 p = 0.0004	LE: -59.343<br>LD: -91.661<br>t= 0.925 p = 0.360

SI 4: Random selection was done by using the case selection tool of the IBM SPSS Statistics 20 package. Factors potentially indicating group differences in attentional bias are highlighted in grey (significant factors are highlighted in red, trends in orange).
